# Lead-Free Piezoelectric Acceleration Sensor Built Using a (K,Na)NbO_3_ Bulk Ceramic Modified by Bi-Based Perovskites

**DOI:** 10.3390/s23021029

**Published:** 2023-01-16

**Authors:** Min-Ku Lee, Byung-Hoon Kim, Gyoung-Ja Lee

**Affiliations:** Smart Sensing and Diagnosis Research Division, Korea Atomic Energy Research Institute, Daejeon 34057, Republic of Korea

**Keywords:** lead-free piezoelectrics, accelerometer, (K,Na)NbO_3_-based ceramics, metamodeling

## Abstract

Piezoelectric accelerometers using a lead-free (K,Na)NbO_3_ (KNN) piezoceramic modified by a mixture of two Bi-based perovskites, Bi(Na,K,Li)ZrO_3_ (BNKLZ) and BiScO_3_ (BS), were designed, fabricated and characterized. Ring-shaped ceramics were prepared using a conventional solid-state reaction method for integration into a compression-mode accelerometer. A beneficial rhombohedral–tetragonal (R–T) phase boundary structure, especially enriched with T phase, was produced by modifying intrinsic phase transition temperatures, yielding a large piezoelectric charge coefficient *d*_33_ (310 pC/N) and a high Curie temperature *T*_c_ (331 °C). Using finite element analyses with metamodeling techniques, four optimum accelerometer designs were obtained with high magnitudes of charge sensitivity *S*_q_ and resonant frequency *f*_r_, as evidenced by two key performance indicators having a trade-off relation. Finally, accelerometer sensor prototypes based on the proposed designs were fabricated using the KNN-BNKLZ-BS ceramic rings, which exhibited high levels of *S*_q_ (55.1 to 223.8 pC/g) and mounted *f*_r_ (14.1 to 28.4 kHz). Perfect charge-to-acceleration linearity as well as broad flat frequency ranges were achieved with excellent reliability. These outstanding sensing performances confirm the potential application of the modified-KNN ceramic in piezoelectric sensors.

## 1. Introduction

Although Pb(Zr,Ti)O_3_ (PZT)-based ceramics have dominated the commercial market of electronic components and devices, including various types of piezoelectric sensors, actuators and ultrasonic transducers [1], their high content of toxic lead (60 wt%) presents serious environmental concerns during preparation and disposal processes. Because there have been global considerations, laws and regulations, spreading from the European Union to many other parts of the world, strongly demanding the elimination of lead-based materials from all consumer items [2,3], there is a pressing need to develop lead-free piezoelectric devices using a viable alternative to PZT.

In efforts to develop nontoxic replacements, both extensive and intensive research activities have been devoted to identifying lead-free piezoceramics during the last couple of decades [4,5,6]. Among potential lead-free families, (K,Na)NbO_3_ (KNN)-based ceramics are, so far at least, widely considered to be promising candidates because of their large piezoelectric activity and high Curie temperature [7,8]. Since the work by Saito, et al. [9], great progress has been made in the physical properties and mechanisms of this material system by chemical doping. This progress has been so substantial that certain electrical properties of novel KNN compositions are comparable to or exceed those of PZT when the ceramics possess a certain phase boundary structure (e.g., rhombohedral–tetragonal (R–T) structure) [10,11,12,13,14]. Although this material system has shown great potential, no single lead-free composition has yet been proposed for piezoelectric devices or components with proven reliability to replace PZT ceramics.

In addition to developing novel materials, the successful application of lead-free compositions requires additional steps, including, in particular, the design, fabrication and performance validation of a piezoelectric device built using a lead-free material system. KNN-based materials are only beginning to find increasing applications, such as in energy harvesting devices [15,16,17,18], actuators [19,20,21,22], ultrasonic transducers [23,24,25,26] and acoustic emission sensors [27,28,29]. To achieve high-performance piezoelectric devices with this material, intensive research efforts on the entire process, from design to fabrication and then validation, are required.

Piezoceramic-based accelerometers are crucial to detect changes in oscillations and vibrations of machines and structures. These sensors are widely used to monitor conditions and safety in various industrial facilities, as well as in aerospace/automotive components [30,31]. Regarding lead-free bulk piezoelectric accelerometers, only a few studies have been made using BNT-based piezoelectric ceramic compositions as another candidate for a lead-free element; examples here include (Bi_1/2_Na_1/2_)TiO_3_-(Bi_1/2_K_1/2_)TiO_3_-BaTiO_3_ (BNKBT) [32] or (Bi_1/2_Na_1/2_)TiO_3_-(Bi_1/2_K_1/2_)TiO_3_-(Bi_1/2_Li_1/2_)TiO_3_-BaTiO_3_ (BNKLBT) systems [33]. Thus, for the first time, this article reports the fabrication of a lead-free, KNN-based bulk piezoelectric accelerometer. In this work, we comprehensively present the design, fabrication and characteristics of a piezoelectric accelerometer built using lead-free, KNN-based ceramics. Polycrystalline, KNN-based ceramic rings, doped with two Bi-containing perovskite oxides, i.e., a 0.03 mole fraction of Bi_0.5_(Na_0.2_K_0.1_Li_0.7_)_0.5_ZrO_3_ (BNKLZ) and 0.01 mole fraction of BiScO_3_ (BS), were prepared as lead-free piezoelectric elements to be integrated into a sensor prototype. The material composition was chosen based on our previous investigation of the effects of these two dopant oxides on the piezoelectric activity and Curie temperature of KNN [34]. Among the piezoelectric transduction modes, the compression mode, where the piezoelectric element performs under compression, was investigated. To obtain accelerometer designs with enhanced sensing performance (sensitivity and resonant frequency), structural designs were examined by numerical simulations, considering the design variables of the constituent components of the accelerometer. Finally, lead-free KNN-BNKLZ-BS accelerometer prototypes were produced using the proposed designs, and their sensing performance was characterized.

## 2. Materials and Methods

The polycrystalline 0.96KNN-0.03BNKLZ-0.01BS ceramic rings to be integrated into a sensor prototype were prepared using the conventional solid-state powder method. The details of preparation conditions are described in Figure S1. The typical dimensions of the ceramic rings were 12.6 mm in outer diameter (O.D.), 7.5 mm in inner diameter (I.D.) and 2.65 mm in thickness. The sintered ceramic rings had densities of 4.25–4.32 g/cm^3^ (greater than 95% of the theoretical density), determined based on the Archimedes method. The poling conditions were determined through pre-tests in silicon oil at room temperature.

The phase boundary structure of the unpoled samples was characterized based on the Rietveld refinement method [34] with an X-ray diffractometer (XRD; D/Max-2500; Rigaku, Tokyo, Japan) using Cu *K*α radiation at a power of 40 kV and 15 mA and at a scan speed of 1°/min. The microstructure was investigated using a field-emission scanning electron microscope (FE SEM; Sirion, FEI, Eindhoven, The Netherlands) with an operating voltage of 20 kV. The dielectric constant *ε*_r_ was measured for the unpoled samples between –150 and 500 °C using two impedance analyzers (HP 4294A; Agilent, Santa Clara, CA, USA, SI 1260; Solartron, Farnborough, UK). Using the unipolar strain curves of the poled samples, the large-signal piezoelectric coefficient *d*_33_^*^ was determined from the ratio of the maximum strain to the peak electric field, *d*_33_^*^ = *S*_max_/*E*_max_. The static piezoelectric coefficient *d*_33_ was also measured at room temperature using a piezo-*d*_33_ meter (ZJ-6B; IACAS, Beijing, China). The polarization-electric field (*P*-*E*) hysteresis loops as well as small-signal piezoelectric coefficient-electric field (*d*_33_-*E*) hysteresis loops were obtained in a temperature range of 30 to 210 °C using a standard Sawyer–Tower measurement circuit (TF Analyzer 2000E; aixACCT Systems GmbH, Aachen, Germany).

**Figure 1:**
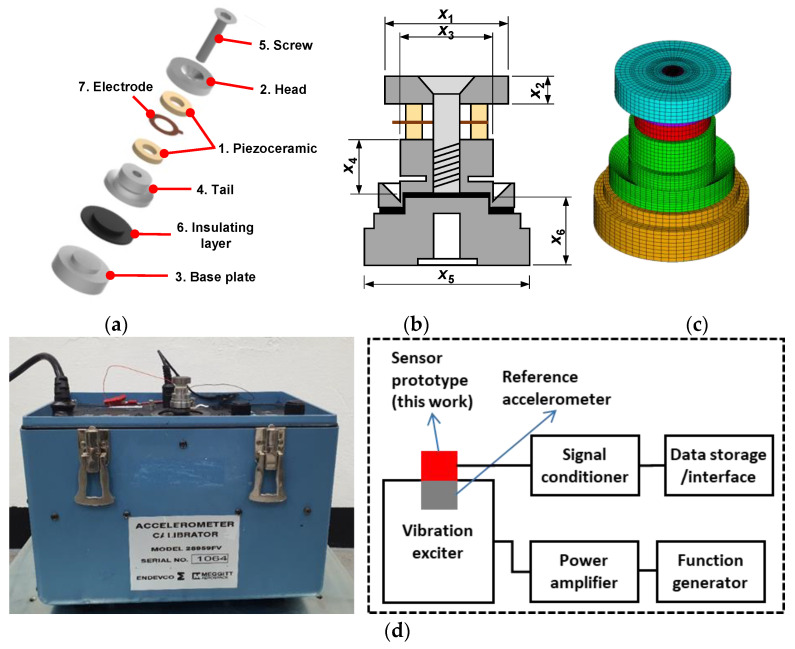
(**a**) Exploded diagram showing the constituent components of the compression-type piezoelectric accelerometer (1: piezoceramic ring; 2: head or seismic mass; 3: base plate; 4: tail; 5: screw; 6: insulating layer; 7: electrode). (**b**) Design variables used for finite element modeling (*x*_1_: head outer diameter, *x*_2_: head height, *x*_3_: tail outer diameter (O.D.), *x*_4_: tail height, *x*_5_: base outer diameter (O.D.), *x*_6_: base height). (**c**) Finite element model of a compression-mode accelerometer (total number of elements: 124,800, number of nodes: 134,977). (**d**) A photo showing experimental setup of the vibration test and its block diagram.

Figure 1a is an exploded diagram showing the structure and arrangement of a typical compression-mode piezoelectric accelerometer sensor with a central preload. The piezoelectric element (1) consists of two piezoceramic rings cut for the longitudinal effect and oriented with their polarities opposite from the central electrode. The two piezoceramic rings are connected electrically in parallel and mechanically in series. They are preloaded under a compressive force between the head (seismic mass) (2) and base plate (3) through the tail (4) by a screw (5). The insulating layer (6) was inserted into the gap between the tail and the base plates. The electrode (7) captures the output signal and feeds it to the connector. When the base plate is accelerated, the seismic mass exerts a proportional force on the piezoceramic element.

Compression-mode accelerometer designs were obtained by numerically optimizing the component design variables related to the head, tail and base plate (Figure 1b). The design and dimensions related to the piezoceramic element were fixed and the same as those of the sintered sample above. Optimization to improve charge sensitivity and resonant frequency characteristics was performed using finite element analysis (FEA) via piezoelectric analysis and metamodeling for free and fixed boundary conditions (Figure 1c). The methodology is described in Supplementary Materials. The required material constants of the constituent components, including those of the KNN-based piezoceramic [35], are presented in Tables S1 and S2.

The accelerometer sensor prototypes were fabricated by assembling the internal sensor components and piezoceramic rings prepared according to the numerically optimized designs (Figure S2). The components such as head, tail and base were manufactured using a CNC (computer numerically controlled) milling machine tool. The specially designed zigs were used for accurate and reliable assembly of the constituent components. The gap between the tail and base was filled with epoxy (ECCOBOND A 359 LV; Emerson & Curming, Germantown, WI, USA) for insulation. In tightening with a screw, the torque value was optimized using a digital torque wrench. The charge sensitivity was evaluated using a portable accelerometer calibrator (28959FV; Endevco, San Juan Capistrano, CA, USA) that includes a built-in vibration exciter, signal generator and computer-controlled amplifier/servo mechanism. The applied acceleration range was between 0.1–10 g (g, gravitational acceleration = 9.8 m/s^2^). The frequency response property of the assembled sensor prototypes was characterized using an impedance analyzer (SI 1260; Solartron Analytical, Farnborough, Hampshire, UK) and a vibration exciter (SE-9; SPEKTRA, Dresden, Germany). The experimental setup for the vibration test is presented in Figure 1d. The sensing performance data of a PZT-based accelerometer were also used for comparison [36].

## 3. Results

### 3.1. Properties of Lead-Free KNN-BNKLZ-BS Ceramic Rings

**Figure 2:**
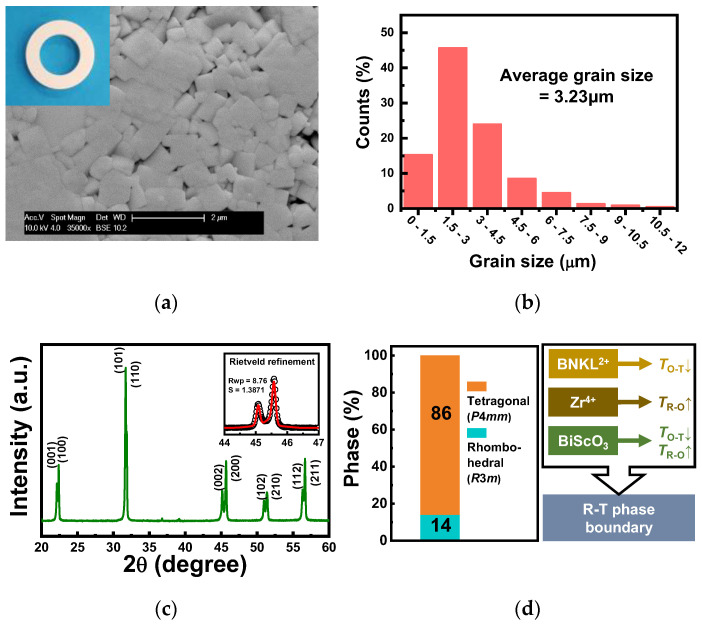
(**a**) Thermally etched SEM image showing the typical microstructure of a sintered KNN-BNKLZ-BS ceramic ring (the inset is a photo of a ceramic ring ready to be assembled into a sensor prototype). (**b**) Grain size distribution. (**c**) Normal θ–2θ XRD pattern (inset is a Rietveld refinement pattern; the low-reliability factor value *R*_wp_ (= 8.76%) as well as the goodness-of-fit indicator *S* (= 1.3871) close to 1 suggest the reliability of the refinement). (**d**) Quantitative phase fraction obtained by Rietveld refinement.

According to SEM images, the KNN-BNKLZ-BS ceramic rings had well-developed perovskite cube grains with clear edges, which is a typical microstructure of KNN systems as a result of sufficient sintering reaction (Figure 2a). The average grain size was estimated to be about 3.23 μm (Figure 2b). The room-temperature XRD pattern revealed that the ceramic rings had a pure perovskite structure without any secondary phases (Figure 2c), confirming a perovskite solid solution induced by the complete diffusion of BNKLZ and BS dopants into the KNN lattice. Based on a Rietveld refinement of the θ-2θ XRD pattern (Figure S3), the resulting ceramic rings revealed a two-phase coexistence comprising R (*R*3 m) and T (*P*4 mm) phases with their respective phase fraction of 14% and 86% (Figure 2d), eventually leading to a T-rich R–T phase boundary structure. It is also known that this R–T phase fraction is adjacent to a condition that causes the maximum piezoelectric response in KNN systems [34,37]. There has been recent progress in the development of KNN systems [10,11,12,13,14]. The construction of an R–T phase boundary at/near room temperature was a major breakthrough in the piezoelectric activity of polycrystalline KNN ceramics, such as the classic morphotropic phase boundary (MPB) in the PZT system. Consequently, co-doping with 3 mol% BNKLZ and 1 mol% BS effectively modified the room-temperature phase structure of orthorhombic (O) KNN by shifting two intrinsic phase transition temperatures, i.e., the R–O transition at −123 °C (*T*_R-O_) and the O–T transition at 210 °C (*T*_O-T_), toward the room temperature region. As also illustrated in Figure 2d, dopants with (Bi,*M*)^2+^ (*M*: alkali metals) and Zr^4+^ ions are known to play effective roles in decreasing *T*_O-T_ and increasing *T*_R-O_, respectively [38,39]. BS is also known to be effective in decreasing *T*_O-T_ and increasing *T*_R-O_ [40,41]. All the changes in phase transitions including T_C_ are related to the distortion of the crystal lattice induced by (Bi,*M*)^2+^ substitution for the A-site and Zr^4+^ for the B-site in KNN [39].

**Figure 3:**
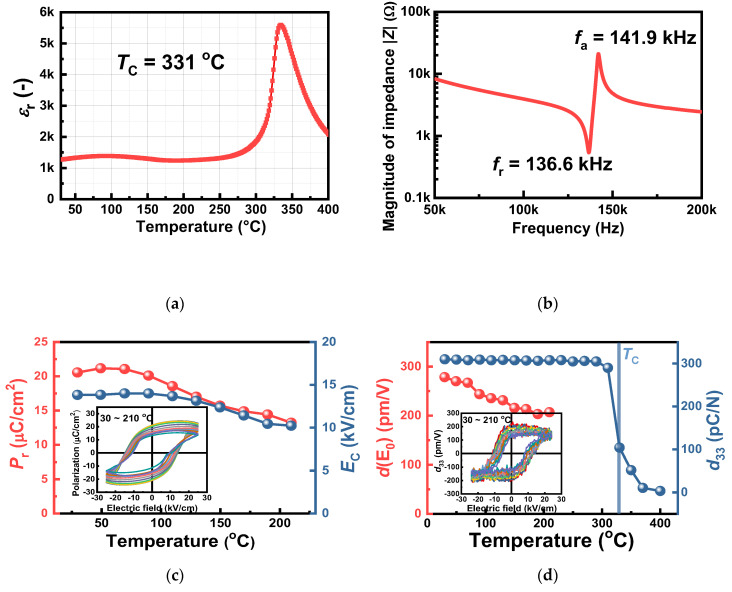
(**a**) Temperature-dependent dielectric constant *ε*_r_ of the KNN-BNKLZ-BS ceramic ring. (**b**) Impedance–frequency profile showing resonant frequency *f*_r_ and anti-resonant frequency *f*_a_. (**c**) Temperature-dependent *P*_r_ and *E*_C_ (inset shows temperature-dependent *P*-*E* loops between 30 and 210 °C). (**d**) In-situ temperature-dependent *d*(E_0_) and thermal aging of static *d*_33_ (inset shows temperature-dependent small-signal *d*_33_-*E* loops between 30 and 210 °C).

The room-temperature values of static *d*_33_ and large-signal *d*_33_^*^ (= *S*_max_/*E*_max_) of the KNN-BNKLZ-BS ceramic ring were measured to be about 310 pC/N and 441 pm/V. The Curie temperature (*T*_c_) determined from the dielectric peak position was 331 °C (Figure 3a). These piezoelectric properties are directly related to the sensitivity and usage temperature for piezoelectric sensing, suggesting that the present KNN composition is practically feasible. The impedance–frequency profile revealed that the anti-resonant (*f*_a_) and resonant frequencies (*f*_r_) were 141.9 and 136.6 kHz, respectively, (Figure 3b) and the electromechanical coupling factor *k*_p_ was determined to be 0.30. Other physical properties are presented in Table 1, including those of the PZT ceramic rings prepared for comparison.

To gain insights into the temperature behavior of the ferroelectric/piezoelectric responses of the KNN-BNKLZ-BS ceramic rings, the *P*-*E* hysteresis loops and field-dependent, small-signal *d*_33_ loops were obtained between 30–210 °C (see the insets of Figure 3c,d). Since the temperature-dependent, small-signal *d*_33_ is considered to be equivalent to the value measured by a quasi-static *d*_33_ meter [42,43], the field-dependent *d*_33_ was measured to investigate the in-situ temperature behavior of piezoelectric activity. Finally, the temperature dependence of remanent polarization *P*_r_, coercive field *E*_c_ and small-signal *d*(E_0_) was obtained from the hysteresis loops, as shown in Figure 3c,d. Here, the *d*(E_0_) was determined to be a positive value in a zero field in the small-signal *d*_33_(E) loops at various temperatures (see the inset). All of the in-situ values for *P*_r_, *E*_c_ and *d*(E_0_) decreased gradually with increasing temperature from 30 to 210 °C. At 110 °C, a 9.9% decrease in *P*_r_ as well as a 15.4% decrease in *d*(E_0_) were observed with respect to their room-temperature values (*P*_r_~20.6 μC/cm^2^, *d*(E_0_)~278.6 pm/V), showing relatively stable temperature behavior. The thermal aging experiments showed that the static *d*_33_ remained unchanged up to about 300 °C, showing excellent thermal stability (with only a 1.9% drop in its room-temperature value), followed by a drastic decrease with a further increase in temperature, owing to the presence of *T*_c_ at around 330 °C. The resulting piezoelectric properties of the present KNN composition showed sufficient potential for use in piezoelectric sensors.

### 3.2. Numerical Simulations of the KNN-Based Piezoelectric Accelerometer Design

**Figure 4:**
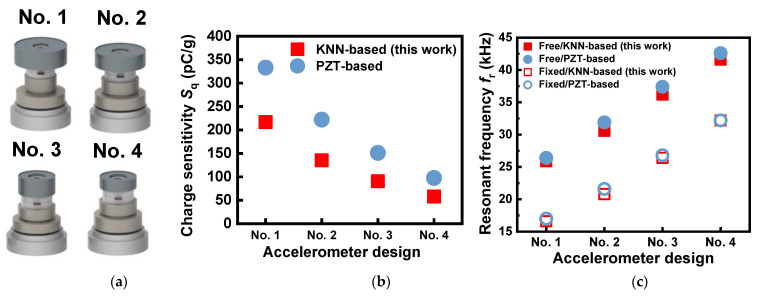
(**a**) Three-dimensional images of the four accelerometer designs (Nos. 1 to 4). (**b**) Numerically calculated results of charge sensitivity *S*_q_ for the four designs built with KNN-BNKLZ-BS piezoelectric elements. (**c**) Numerically calculated results of resonant frequency *f*_r_ of the four designs under free and fixed modes. Numerically analyzed results for PZT-based accelerometers [36] are also compared in (**b**,**c**).

Figure 4a shows the three-dimensional images of four accelerometer designs obtained by numerical simulations. The designs numbered 1 to 4 were characterized using different dimensions of the constituent components (Table 2). By using the material data of the KNN-based ceramics [35], we could obtain numerically analyzed results for charge sensitivity *S*_q_ and resonant frequency *f*_r_ for the optimized designs, as shown in Figure 4b,c. It can be seen that the *S*_q_ value, which is related to the sensitivity of the accelerometer, decreased from 216.8 pC/g to 57.9 pC/g, when the design changed from No. 1 to No. 4. The most influential factors among the investigated design variables were the head’s O.D. (*x*_1_) and height (*x*_2_). The observed variation was thus found to be associated with the weight of the head, which is proportional to the *S*_q_ property.

For the four optimized designs, the impedance–frequency characteristics obtained from the numerical simulations of the free and fixed boundary conditions are presented in Figure S4. The *f*_r_ values for the fixed mode were lower by about 9.3–9.8 kHz compared to those for the free mode. Unlike the trend in *S*_q_, the *f*_r_ value for both the free and fixed modes increased almost linearly, as the design changed from No. 1 to No. 4. The *f*_r_ value for the fixed mode, which corresponds to a mounted or attached condition in a practical sensor system, increased from 16.6 to 32.2 kHz, and these levels of *f*_r_ were found to be in the range of those of commercial piezoelectric accelerometers. From Figure 4b,c, the correlation between *S*_q_ and *f*_r_ was found to be negative, that is, a higher *S*_q_ resulted in a lower *f*_r_. Hence, the two performance indicators have a trade-off relation.

When compared with the numerically analyzed results of the PZT-based accelerometer, the present *S*_q_ values of the KNN-based prototype were lower, mostly resulting from the effect of reduced piezoelectric activity; the *d*_33_ values used in the numerical work were 321 pC/N for KNN and 400 pC/N for PZT. However, the *f*_r_ values obtained from the KNN-based accelerometer prototype were almost equivalent to those of the PZT-based one. This indicates that the *f*_r_ property is not affected by the piezoelectric activity of the piezo element but by structural design factors (dimensions and configuration) or the mechanical properties of the components.

### 3.3. Characterization of Accelerometer Prototype Built Using Lead-Free KNN-BNKLZ-BS Ceramics

**Figure 5:**
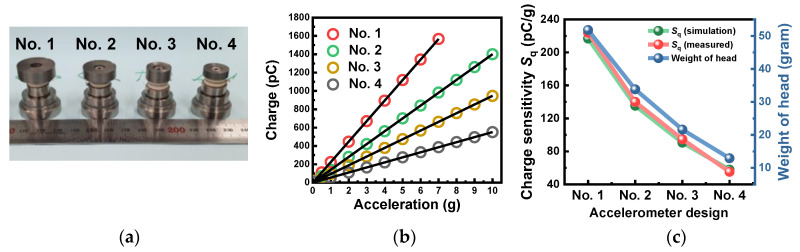
(**a**) Photos of accelerometer prototypes fabricated using the optimized designs (Nos. 1 to 4). (**b**) Charge versus acceleration measured for the corresponding accelerometer prototypes (test frequency = 159 Hz). (**c**) Comparison of the numerically analyzed charge sensitivity *S*_q_ and an experimentally measured value.

Using the designs obtained from the numerical work, four kinds of accelerometer prototypes were fabricated using KNN-BNKLZ-BS ceramics (Figure 5a), and their charge-to-acceleration characteristics were measured by a portable accelerometer calibrator, as shown in Figure 5b. In the investigated acceleration ranges, the values of Pearson’s correlation coefficient *r* were almost unity for all of the sensor prototypes, revealing a perfect linear response between the charge and acceleration. These outstanding results prove the high reliability of the assembled sensor prototypes. The slopes, representing a charge sensitivity *S*_q_, were determined from their linear relation (Figure 5c and Table 3). The measured *S*_q_ decreased from 223.8 to 55.1 pC/g, as the accelerometer design changed from No. 1 to No. 4. By considering the piezoceramic and seismic mass (head), the *S*_q_ was simplified and is given by Equation (1) [44].

*S*_q_ = *n*⋅*d*_33_⋅*m*_s_⋅*g* (1)

Here, *n*, *m*_s_ and *g* are the number of piezoceramic layers, the weight of seismic mass and acceleration, respectively. According to the numerically analyzed *S*_q_ property, the design factor most affecting sensitivity was the head. In a real system, likewise, the weight of the head positively affected charge sensitivity with strong correlation (Figure 5c).

**Figure 6:**
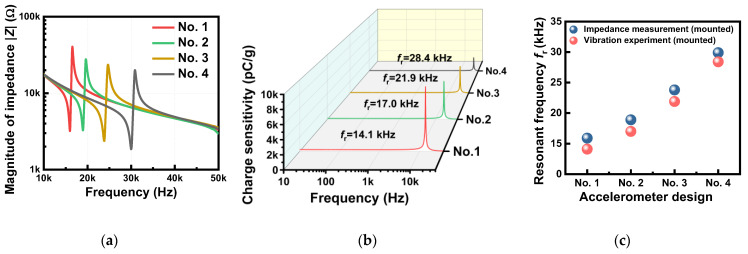
(**a**) Impedance–frequency spectra measured from impedance measurements for accelerometer prototypes in the mounted condition. (**b**) Frequency response profiles measured from vibration experiments in the mounted condition. (**c**) Results of resonant frequency *f*_r_ determined from both measurements.

Next, impedance tests and vibration experiments were performed to investigate the frequency response and *f*_r_ properties. Figure 6a,b show the impedance–frequency spectra measured in the free and fixed condition and the frequency response profiles obtained from the vibration experiments in the fixed condition, respectively, for four different accelerometer prototypes made of KNN-BNKLZ-BS ceramic rings. The properties of anti-resonant frequency *f*_a_, including impedance *Z*, are also presented in Figure S5. In the impedance measurement, the *f*_r_ was found to vary from 15.9 to 29.9 kHz, depending on the design. The measured *f*_r_ values were found to be almost similar to those obtained from the vibration experiment, with only a slight difference (1.5 to 1.9 kHz). The resonant frequency *f*_r_ of the piezoelectric accelerometer was simplified and is given by Equation (2) [45].

(2) $$f_{r}=\frac{1}{2\pi }\cdot {\left(\frac{k}{m_{s}}\right)}^{\frac{1}{2}}=\frac{1}{2\pi }\cdot {\left(\frac{E_{33}\cdot \pi \cdot D_{p}{}^{2}}{4\cdot n\cdot m_{s}\cdot t_{p}}\right)}^{\frac{1}{2}}$$

Here, *D*_p_, *t*_p_, *k* and *E*_33_ are the diameter, thickness, stiffness and Young’s modulus of the piezoceramic, respectively. From this relation, it is known that the weight of the head (*m*_s_) negatively affects the resonant frequency *f*_r_; $f_{r}\propto 1/\sqrt{m_{s}}$. Therefore, the lower *m*_s_ leads to an increase in *f*_r_, as the design changes from No. 1 to No. 4, consistent with the above numerical results. This negative correlation of *f*_r_ with *m*_s_ demonstrates there is an inverse relationship between the two key sensing performance metrics of sensitivity and resonant frequency. Since the design variables can only improve one of the two performance metrics while sacrificing the other, a design can be chosen to enhance either the sensitivity or the resonant frequency for vibration sensing. We also note that the experimentally measured *f*_r_ values were lower than the numerically calculated values (Figure 4c): in the fixed condition, their differences were 2.5 to 4.5 kHz. These discrepancies between experimental results and numerical predictions might be related to loose boundaries or interfaces inside the real system, while the numerical analysis assumed perfect contacts between the internal components. It is also believed that this is related to the difficulties in uniformly controlling the epoxy layer thickness relative to the *f*_r_.

From a practical standpoint, it is extremely important that the piezoelectric accelerometer has a large flat frequency range, as this extends its usable detection range below a critical point of *f*_r_ [1,46]. The flatness was determined from the frequency response profiles obtained from the vibration experiments, as presented in Table 4. The amplitude deviations were employed as the performance criteria, based on the requirements used in commercial sensors, which were ±5%, ±10% and ±3 dB in the amplitude-frequency curves [46,47]. The flat response range, determined from Figure 6b, became broader as the mounted *f*_r_ increased. The observed *f*_r_-dependent flat frequency ranges were substantially high compared to those of commercial PZT accelerometers [47], proving the reliability of the modified KNN-based accelerometer prototypes.

**Figure 7:**
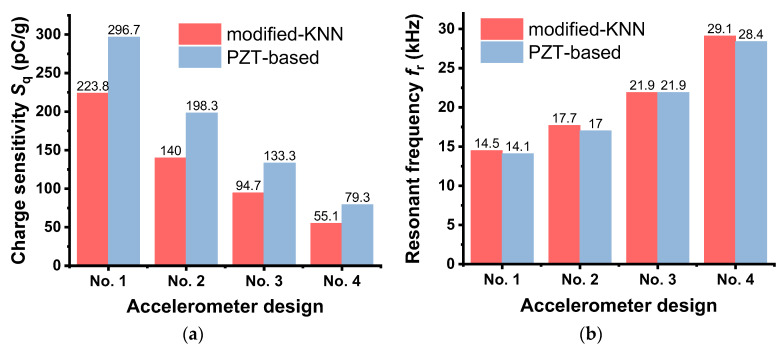
Comparison of (**a**) charge sensitivity *S*_q_ and (**b**) resonant frequency *f*_r_ of accelerometer prototypes made of KNN-BNKLZ-BS and PZT ceramics. Experimentally measured results for the PZT-based accelerometers were taken from ref. [36].

Figure 7 shows the comparison of *S*_q_ and *f*_r_ for the accelerometer prototypes made of the modified-KNN and PZT ceramics. The *S*_q_ values obtained from the KNN-based accelerometers were 24.6 to 30.5% lower than those of the PZT-based accelerometers, similar to the numerically analyzed results above (Figure 4b). This primarily results from the effect of *d*_33_ (310 pC/N), which is lower than that of PZT (400 pC/N), considering the relation $S_{q}\propto d_{33}$ in Equation (1). In contrast, the *f*_r_ properties of the KNN-based accelerometers were at nearly the same level as those of the PZT-based accelerometer. When compared to BNT-based accelerometers [32,33], the sensing performance of the present KNN-based accelerometer was excellent. For example, the charge sensitivity value of the BNT-based sensor was as low as 29.1 or 42.6 pC/g at frequencies ranging from 50 Hz to 8.24 kHz, while the KNN-based accelerometer showed a much greater sensitivity of 223.8 pC/g within a similar frequency range (10 Hz to 8.0 kHz). Consistent with the numerical results in Figure 4c, the experimentally measured data also revealed that *f*_r_ is a function of the mechanical properties of the materials and the design parameters (Equation (2)), not piezoelectric activity.

## 4. Conclusions

The design, fabrication and sensing characteristics of a piezoelectric accelerometer built using lead-free, KNN-based ceramics were comprehensively investigated. Polycrystalline KNN-based ceramic rings, specially modified by doping with BNKLZ and BS, were prepared for integration into a compression-mode accelerometer. The sintered ceramic rings showed a T-rich R–T phase boundary structure (a mixture of 86% T phase and 14% R phase) with a large *d*_33_ of 310 pC/N and a high *T*_c_ of 331 °C. Four compression-mode accelerometer designs and their resulting *S*_q_ and *f*_r_ properties were obtained by numerically optimizing the structural designs of the sensor components, including head (seismic mass), tail and base plate. Finally, the accelerometer sensor prototypes were fabricated using the proposed designs and KNN-BNKLZ-BS ceramic rings. They exhibited excellent sensing performance with high levels of *S*_q_ (55.1 to 223.8 pC/g) and mounted *f*_r_ (14.1 to 28.4 kHz). Moreover, there was a perfect linearity between charge and acceleration, with a Pearson’s correlation coefficient *r* close to 1 for all of the assembled sensor prototypes. The experimentally measured flat frequency response ranges were significantly high and comparable to those of commercial PZT accelerometers. The trade-off relationship between the *S*_q_ and *f*_r_ was also experimentally confirmed, consistent with the numerically analyzed results. In light of these desirable features, the present modified-KNN ceramic has practical potential for applications in lead-free piezoelectric sensors.

## Figures and Tables

**Table 1:** Material properties of KNN-BNKLZ-BS and PZT ceramic rings.

Material Property	KNN-BNKLZ-BS	PZT (Ref. [36])
Weight *m* (g)	0.809	1.616
Density *ρ* (kg/m^3^)	4101.2	7767.7
Outer diameter O.D. (mm)	12.40	12.62
Inner diameter I.D. (mm)	7.46	7.52
Thickness *t* (mm)	2.56	2.57
Remanent polarization *P*_r_ (μC/cm^2^)	20.5	15.9
Coercive field *E*_C_ (kV/cm)	13.8	14.7
Dielectric constant *ε*_r_	1530	1850
Loss factor tanδ	0.03	0.02
Electromechanical coupling factor *k*_p_	0.30	0.36
Mechanical quality factor *Q*_m_	72	81
Large-signal piezoelectric coefficient *d*_33_^*^ (pm/V)	441.1	726.2
Static piezoelectric coefficient *d*_33_ (pC/N)	310.0 ± 4.8	400.0 ± 2.1
Curie temperature *T*_C_ (°C)	331	367

**Table 2:** Values of optimized design variables (*x*_1_ to *x*_6_) for the four accelerometer designs and numerically analyzed charge sensitivity *S*_q_ and resonant frequency *f*_r_ for the free and fixed boundary conditions.

Design no.	*x*_1_ (mm)	*x*_2_ (mm)	*x*_3_ (mm)	*x*_4_ (mm)	*x*_5_ (mm)	*x*_6_ (mm)	Free Mode		Fixed Mode	
							*f*_r_ (kHz)	*S*_q_ (pC/g)	*f*_r_ (kHz)	*S*_q_ (pC/g)
1	23	7	13.94	5.68	26.4	2.35	25.9	216.8	16.6	216.8
2	20.5	5.75	15.1	4.91	24.4	3.2	30.6	135.3	20.8	135.3
3	15.08	7	15.1	4.53	24.4	1.63	36.2	90.5	26.4	90.5
4	15	4.28	15.2	4.78	24.4	3.03	41.6	57.9	32.2	57.9

**Table 3:** Acceleration range, Pearson’s correlation coefficient, charge sensitivity and weight of head for the four accelerometer prototypes.

Design No.	Acceleration Range (g)	Pearson’s Correlation Coefficient *r*	Charge Sensitivity *S*_q_ (pC/g)	Weight of Head *m*_s_ (gram)
1	0.1–7	1	223.8 ± 0.1	51.8
2	0.1–10	1	140.0 ± 0.1	33.8
3	0.1–10	1	94.7 ± 0.03	21.6
4	0.1–10	0.9999	55.1 ± 0.04	12.9

**Table 4:** Frequency response properties of the accelerometer prototypes obtained from vibration experiments.

Design No.	Frequency Response			Mounted *f*_r_ (kHz)
	±5%	±10%	±3 dB	
1	10 Hz to 4.2 kHz	10 Hz to 5 kHz	10 Hz to8.0 kHz	14.1
2	10 Hz to 5.1 kHz	10 Hz to 6.1 kHz	10 Hz to 9.6 kHz	17.0
3	10 Hz to 6.9 kHz	10 Hz to 8.3 Hz	10 Hz to 12.7 kHz	21.9
4	10 Hz to 7.1 Hz	10 Hz to 10.9 kHz	10 Hz to 16.6 kHz	28.4

## Data Availability

Not applicable.

## Supplementary Materials

The following supporting information can be downloaded at https://www.mdpi.com/article/10.3390/s23021029/s1: Table S1: Component materials and their mechanical properties (density, Young’s modulus and Poisson’s ratio); Table S2: Electrical and mechanical properties of KNN-based piezoceramic used for numerical simulation [34]; Figure S1: Preparation conditions of KNN-0.03BNKLZ-0.01BS ceramic rings by conventional solid-state powder method; The methodology of design optimization: the related references [48,49,50,51,52] are cited from the supplementary materials; Figure S2: Fabrication procedures of a compressive-type piezoelectric accelerometer prototype using KNN-BNKLZ-BS ceramic rings and sensor components: a specially designed zig was used for reliably aligning each sensor component, and an injection mold of our own design was also used to precisely control the amount of epoxy to be injected; Figure S3: Rietveld refinement of the XRD pattern of the KNN-003BNKLZ-0.01BS ceramic ring. Results of cell parameters, phase fraction and reliability factors (*R*_wp_ and *S*) are listed in the table; Figure S4: Impedance–frequency spectra and values of *f*_r_, *f*_a_ and *Z* obtained from numerical simulation using free and fixed boundary conditions for KNN-based accelerometer designs (Nos. 1 to 4); Figure S5: Impedance–frequency profiles and values of resonant and anti-resonant frequencies (*f*_r_ and *f*_a_) and magnitude of impedance ∣*Z*∣ experimentally measured under free and fixed conditions from impedance tests for KNN-based accelerometer prototypes (design Nos. 1 to 4).
