# Supplementary material for: Lead-Free Piezoelectric Acceleration Sensor Built Using a (K,Na)NbO3 Bulk Ceramic Modified by Bi-Based Perovskites
Source: Sensors (Basel). 2023 Jan 16;23(2):1029. doi: 10.3390/s23021029 (PMC9867131; doi:10.3390/s23021029)
Supplement: Supplementary file 1 [file sensors-23-01029-s001.zip › sensors-2117260-supplementary.pdf]

Supplementary Information

Lead-Free Piezoelectric Acceleration Sensor Built Using a $(K,Na)NbO_3$ Bulk Ceramic Modified by Bi-Based Perovskites

Min-Ku Lee, Byung-Hoon Kim and Gyoung-Ja Lee *

Smart Sensing and Diagnosis Research Division, Korea Atomic Energy Research Institute, Daejeon 34057, Republic of Korea

* Correspondence: leegj@kaeri.re.kr; Tel.: +82-42-868-4838

Table S1. Component materials and their mechanical properties (density, Young's modulus, and Poisson's ratio).

Component	Material	Density (kg/m^3)	Young's modulus (GPa)	Poisson's Ratio (-)
Head	Tungsten	17,900	385	0.2
Tail	316 stainless steel	7767	193	0.29
Base	316 stainless steel	7767	193	0.29
Insulating layer	Epoxy	1400	9.5	0.35

Table S2. Electrical and mechanical properties of KNN-based piezoceramic used for numerical simulation [34].

Physical properties		Piezoelectric coefficient (pC/N)			Dielectric constant	Elastic compliance (10^{-12} m^2/N)					
Density (kg/m^3)	Poisson's ratio	d_{31}	d_{33}	d_{15}	$\varepsilon_{33}/\varepsilon_o$[1]	S_{11}^E	S_{12}^E	S_{13}^E	S_{33}^E	S_{44}^E	S_{66}^E
4100	0.3	−159	321	602	523	12.8	−4.42	−5.21	12.3	40.9	34.4

[1]$\varepsilon_o = 8.85 \times 10^{-12}$ F/m

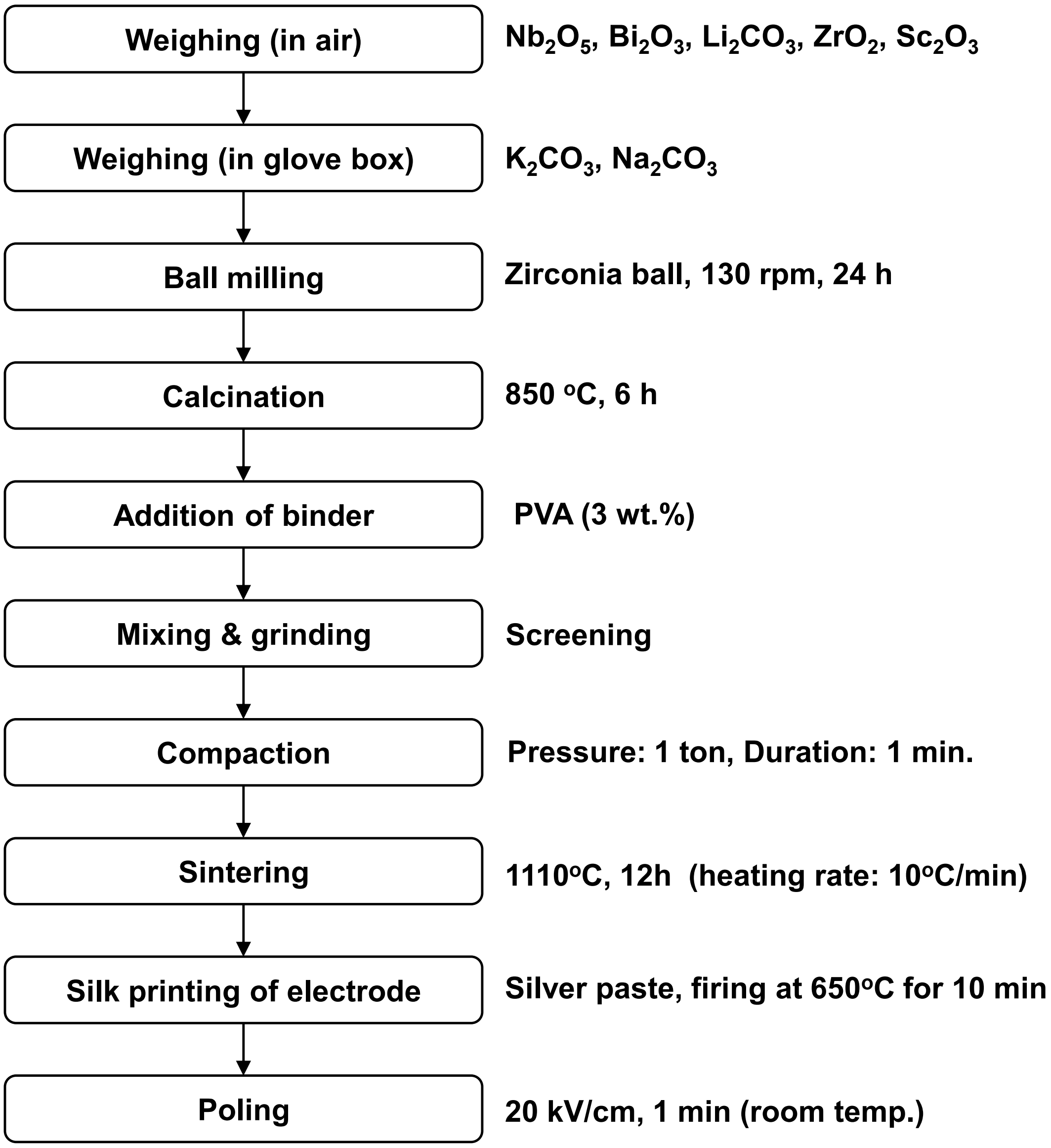

Figure S1. Preparation conditions of KNN-0.03BNKLZ-0.01BS ceramic rings by conventional solid-state powder method.

[The methodology of design optimization]

The finite element analysis (FEA) was performed using a commercial finite element program (ANSYS V18). The total number of elements was 124,800 and the number of nodes was 134,977. Design of experiments (DOE) techniques [48,49] were used to carry out simulation experiments efficiently. The values of the resonant frequency and charge sensitivity were numerically calculated by piezoelectric analysis using the DOE test points. The total number of DOE test points was 201. Using the DOE test points, we carried out metamodeling, which is known as more efficient than the actual analytical model [50,51], to approximate the relationship between the design variables (input) and the performances (output). Since the sensor in this work includes many geometrical design variables and nonlinear characteristics, the kriging model [52], which is a global approximation model, was adopted as a metamodel. Using the kriging model, ten design variables were optimized by a program called Easy Design. The optimization was conducted to find the optimal values of the design variables (x1 to x10) by which the charge sensitivity could be maximized at different resonant frequencies ranging from 20 to 50 kHz. Here, the charge sensitivity was obtained using a sine-wave acceleration of 1 g at 159 Hz.

48. Ross, P. *Taguchi Techniques for Quality Engineering*; McGraw-Hill: New York, NY, USA, 1996.
49. Montgomery, D.C. *Design Analyses of Experiments*, 3rd ed.; John Wiley & Sons: New York, NY, USA, 1997.
50. Kumar, V. Optimization and modelling of process parameters involved in ultrasonic machining of glass using design of experiments and regression approach. *Am. J. Mater. Eng. Technol.* **2013**, *1*, 13–18.
51. Kumar, J.P.N.; Kumar, S.J.; Jeyathilak, R.K.S.; Venkatesh, M.; Christopher, A.S.; Ganesh, K.C. Effect of design parameters on the static mechanical behavior of metal bellows using design of experiment and finite element analysis. *Int. J. Interact. Des. Manuf.* **2017**, *11*, 535–545.
52. Huang, D.; Allen, T.T.; Notz, W.I.; Zeng, N. Global optimization of stochastic black-box systems via sequential Kriging meta-models. *J. Glob. Optim.* **2006**, *34*, 441–466.

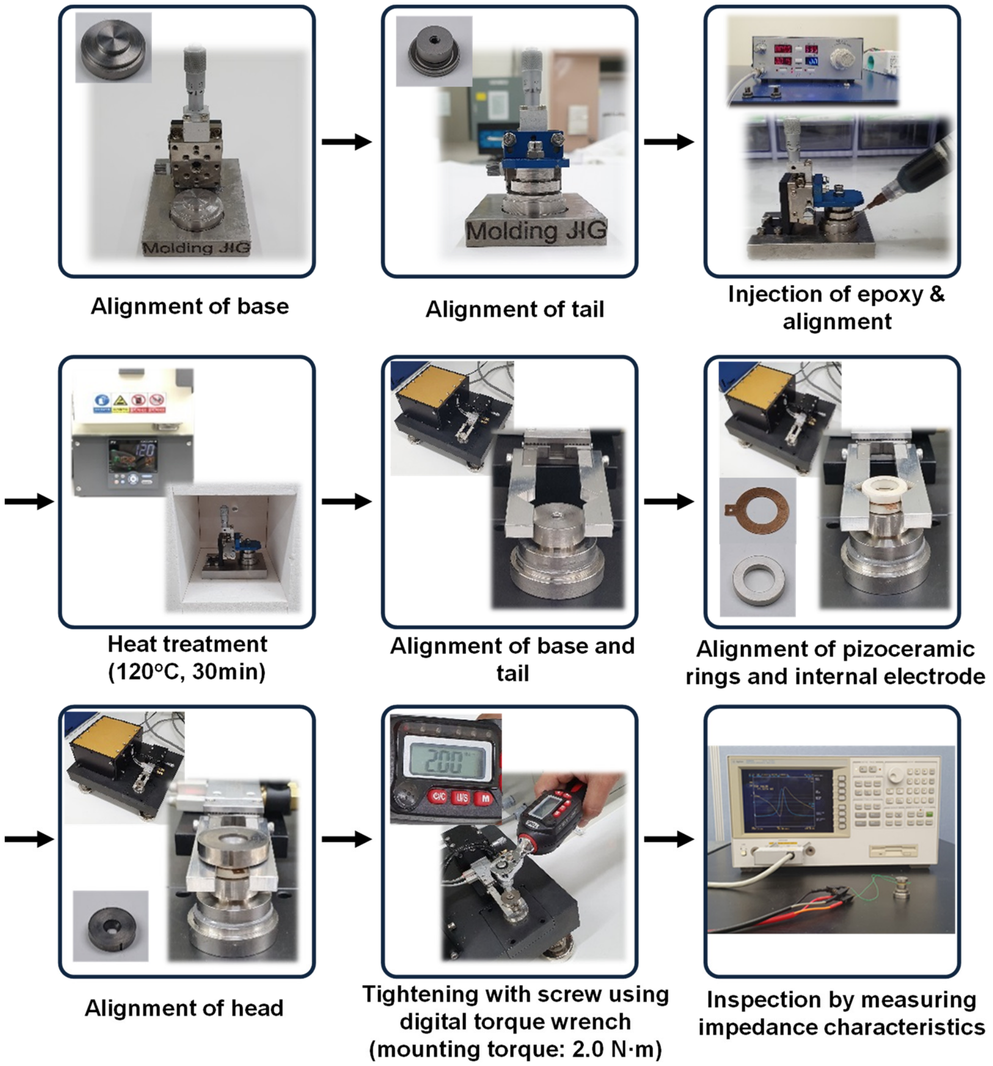

Figure S2. Fabrication procedures of a compressive-type piezoelectric accelerometer prototype using KNN-BNKLZ-BS ceramic rings and sensor components: specially designed zig was used for reliable alignments of each sensor component and an injection mold of our own design was also used to precisely control the amount of epoxy to be injected.

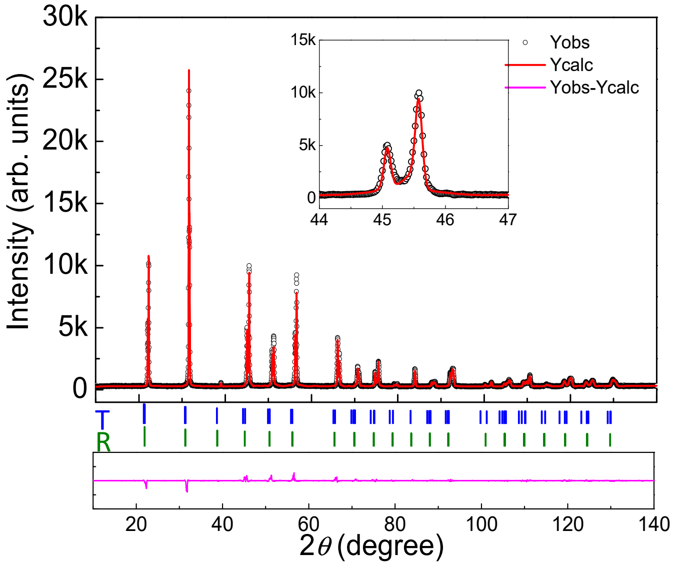

Sample	KNN-0.03BNKLZ-0.01BS
Space group	*R*3*m*<br>*P*4*mm*
Cell parameter for *R*3*m* space group (Å)	3.9783<br>3.9783<br>3.9783
Cell parameter for *P*4*mm* space group (Å)	3.9716<br>3.9716<br>4.0134
Tetragonal phase (%)	86.1
Rhombohedral phase (%)	13.9
Reliability factor R_{wp} (%)	8.76
Goodness-of-fit indicator *S*	1.3871

Figure S3. Rietveld refinement of the XRD pattern of the KNN-003BNKLZ-0.01BS ceramic ring. Results of cell parameters, phase fraction and reliability factors (R_{wp} and *S*) are listed in the table.

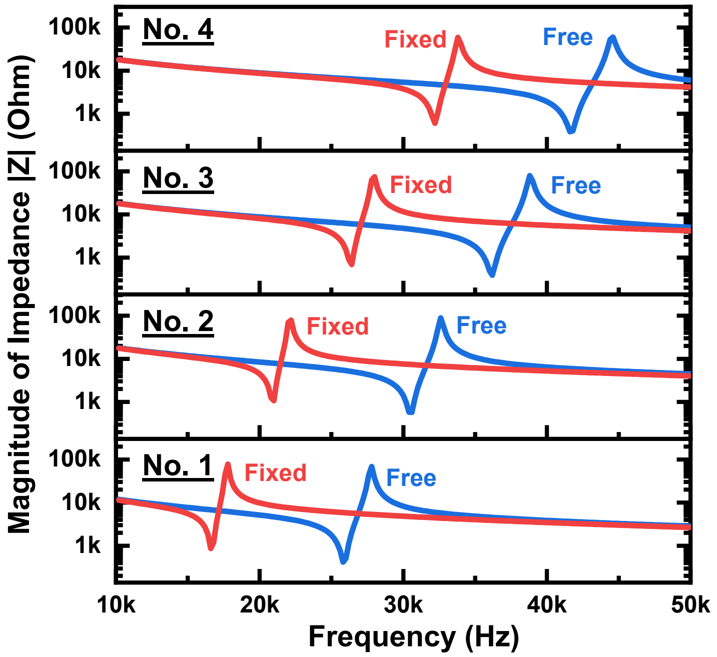

Design no.	Condition	f_r (kHz)	$\lvert Z_r\rvert$ (Ω)	f_a (kHz)	$\lvert Z_a\rvert$ (Ω)
1	Free	25.9	419.49	27.8	68868.7
	Fixed	16.6	864.719	17.8	78358
2	Free	30.6	578.072	32.6	88682.5
	Fixed	20.8	1824.13	22.2	50671.3
3	Free	36.2	393.283	38.8	79289.8
	Fixed	26.4	697.379	28.8	74600.5
4	Free	41.6	385.193	44.6	59731.6
	Fixed	32.2	604.759	33.8	58709.4

Figure S4. Impedance-frequency spectra and values of f_r, f_a and Z obtained from numerical simulation using free and fixed boundary conditions for KNN-based accelerometer designs (nos. 1 to 4).

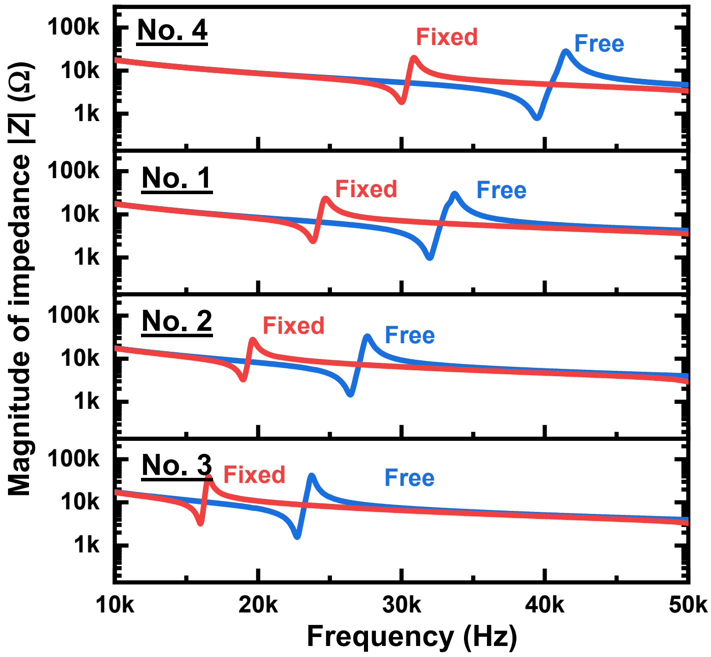

Design no.	Condition	f_r (kHz)	$\lvert Z_r\rvert$ (Ω)	f_a (kHz)	$\lvert Z_a\rvert$ (Ω)
1	Free	22.7	1563.7	23.7	42172
	Fixed	15.9	3192.4	16.5	40519
2	Free	26.4	1449.7	27.6	33332
	Fixed	18.9	3265.5	19.6	27717
3	Free	32.0	966.27	33.7	30440
	Fixed	23.8	2375.2	24.7	23545
4	Free	39.4	780.24	41.5	28512
	Fixed	29.9	1848.7	30.9	19989

Figure S5. Impedance-frequency profiles and values of resonant and anti-resonant frequencies (f_r and f_a) and magnitude of impedance $|Z|$ experimentally measured under free and fixed conditions from impedance tests for KNN-based accelerometer prototypes (design nos. 1 to 4).
